# A Case of Spindle Cell Squamous Cell Carcinoma Manifesting in the Mandible Following Resection of Buccal Mucosal Squamous Cell Carcinoma

**DOI:** 10.7759/cureus.51191

**Published:** 2023-12-27

**Authors:** Shohei Seta, Yoshihide Ota, Hisashi Kato, Yasuhiro Nakanishi

**Affiliations:** 1 Oral Surgery, Ota Memorial Hospital, Ota, JPN; 2 Oral and Maxillofacial Surgery, Tokai University School of Medicine, Kanagawa, JPN

**Keywords:** buccal mucosal squamous cell carcinoma, transformation, mandible, intraosseous carcinoma, spindle cell squamous cell carcinoma

## Abstract

Spindle cell squamous cell carcinoma (SCSCC) represents a distinctive subtype of squamous cell carcinoma, characterized by a marked malignancy and sarcomatoid transformations predominantly comprising spindle-shaped cells. In this context, we executed a surgical resection of a buccal mucosal squamous cell carcinoma, encompassing the mandibular periosteum, for a case where buccal mucosal cancer had pervaded the mandibular gingival mucosa. Notably, in a period of one year and four months subsequent to this procedure, a spindle cell squamous cell carcinoma emerged as an intraosseous carcinoma, originating from the periosteum resection. This report delineates the occurrence of this rare pathology. The subject of this case is an 83-year-old female. She underwent a resection of a buccal mucosal squamous cell carcinoma, including the mandibular gingival periosteum, for cancer on the right buccal mucosa. The histopathological evaluation post-surgery confirmed the diagnosis of squamous cell carcinoma with clear margins. A computed tomography (CT) scan, conducted one year and four months postoperatively, disclosed a contrast-enhanced tumorous growth in the mandible. Owing to the considerable restriction in opening caused by scarring and the attendant challenges in biopsy acquisition, an expedited intraoperative diagnosis was rendered. This preliminary assessment indicated a spindle cell sarcoma, leading to a hemimandibular resection. The final histopathological diagnosis was spindle cell squamous cell carcinoma. Twelve months have elapsed since the surgical intervention, with no evidence of recurrence or metastasis observed to date.

## Introduction

Spindle Cell Squamous Cell Carcinoma (SpC-SCC) is identified as a pernicious variant of squamous cell carcinoma, typified by its highly malignant nature and the manifestation of sarcomatoid changes, primarily characterized by spindle-shaped cells. While numerous instances of SpC-SCC post-radiation therapy or chemotherapy for squamous cell carcinoma have been documented [1-5], the emergence of SpC-SCC subsequent to surgical resection remains a notably rare phenomenon [6]. In this report, we present an exceptional case where partial resection of the buccal mucosa, inclusive of the mandibular periosteum, was necessitated due to the invasion of buccal mucosal cancer into the mandibular gingival mucosa. Remarkably, SpC-SCC was observed to develop within the mandible 1 year and 4 months following this surgical intervention. From my clinical experience, this case merits detailed reporting due to its rarity and the significant implications it holds in oncological surgery.

## Case presentation

An 83-year-old female patient presented at our institution with discomfort in her right buccal mucosa first noticed in April 2020. An initial assessment at a local dental clinic raised suspicions of a tumorous lesion, prompting referral for specialized evaluation. The patient's medical history included hypertension, hyperlipidemia, and osteoporosis, with no relevant familial medical history contributing to her current condition.

During the initial examination, the patient appeared moderately built and well-nourished, standing at a height of 145.9 cm and weighing 51.8 kg. Her Performance Status was rated at 0, with a Karnofsky Performance Status of 100. No external tumor indications were observed in the extraoral examination. The cervical lymph nodes were neither swollen nor tender, and no motor or sensory deficits were detected in the face or neck.

**Figure 1:**
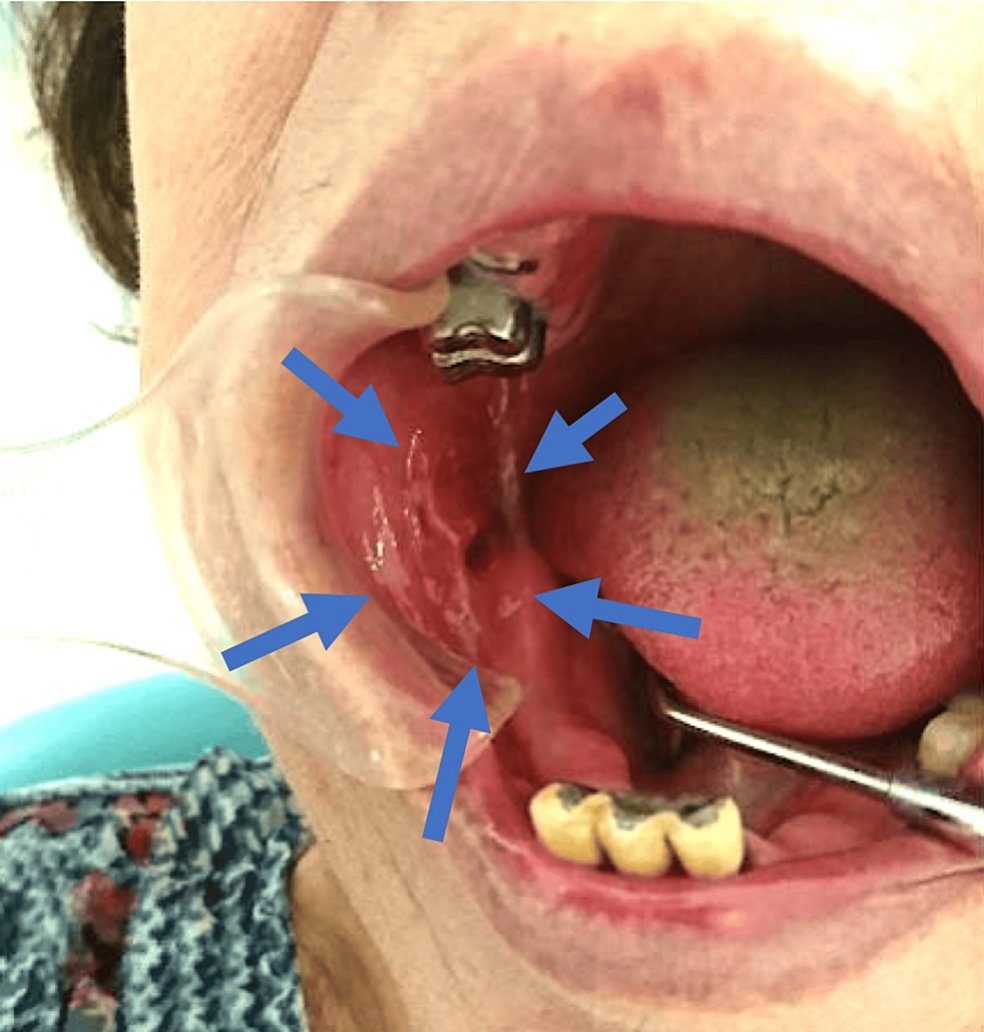
Initial Intraoral Examination A granular ulcerative lesion, measuring 17x15 mm, was observed extending from the right cheek mucosa to the mandibular gingival mucosa.

The intraoral examination revealed a superficial, granular, ulcerative lesion approximately 17 x 15 mm in size, extending from the right buccal mucosa to the mandibular gingival mucosa (Figure 1). Notably, the right lower molar tooth had been extracted over a decade earlier.

**Figure 2:**
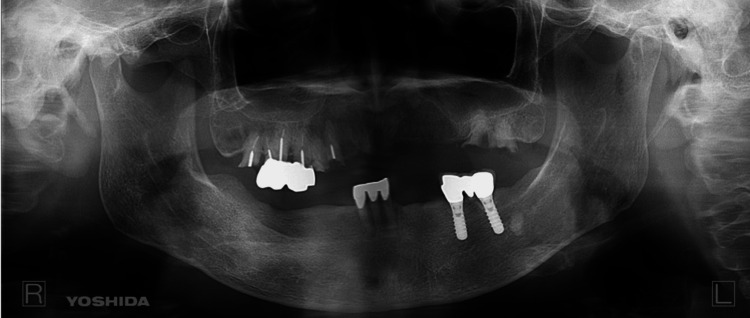
Panoramic X-Ray The panoramic X-ray images displayed no abnormal findings.

Comprehensive diagnostic imaging was undertaken. Panoramic X-ray results showed no discernible abnormalities (Figure 2).

**Figure 3:**
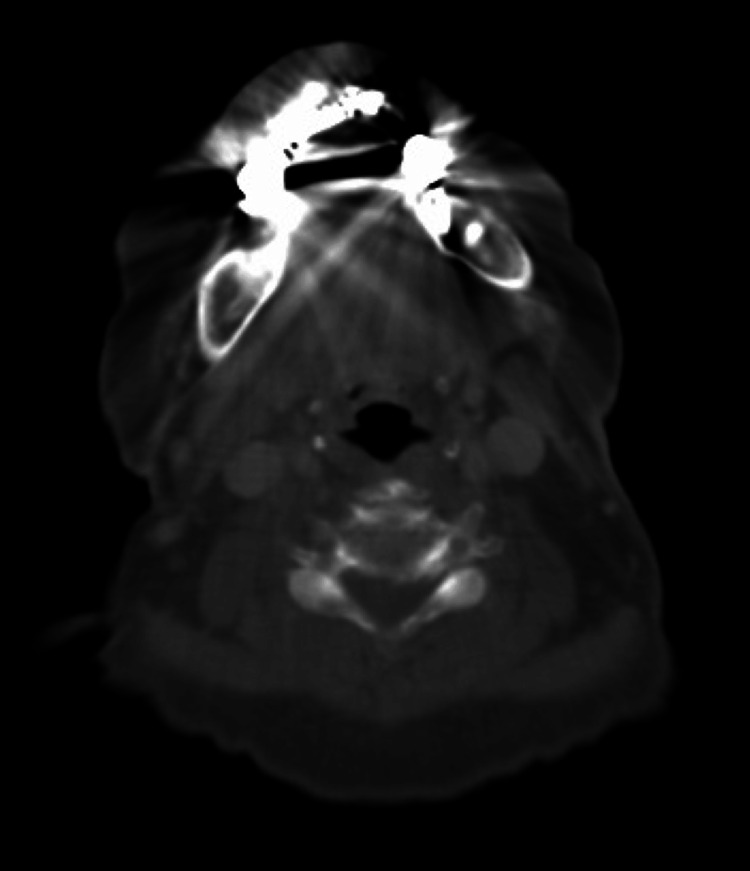
CT Scan Absence of lesions in the right buccal mucosa and no evidence of bone resorption due to tumor. No abnormalities in the neck or distant organs.

The CT scan indicated an absence of lesions in the right buccal mucosa and no signs of bone resorption (Figure 3).

**Figure 4:**
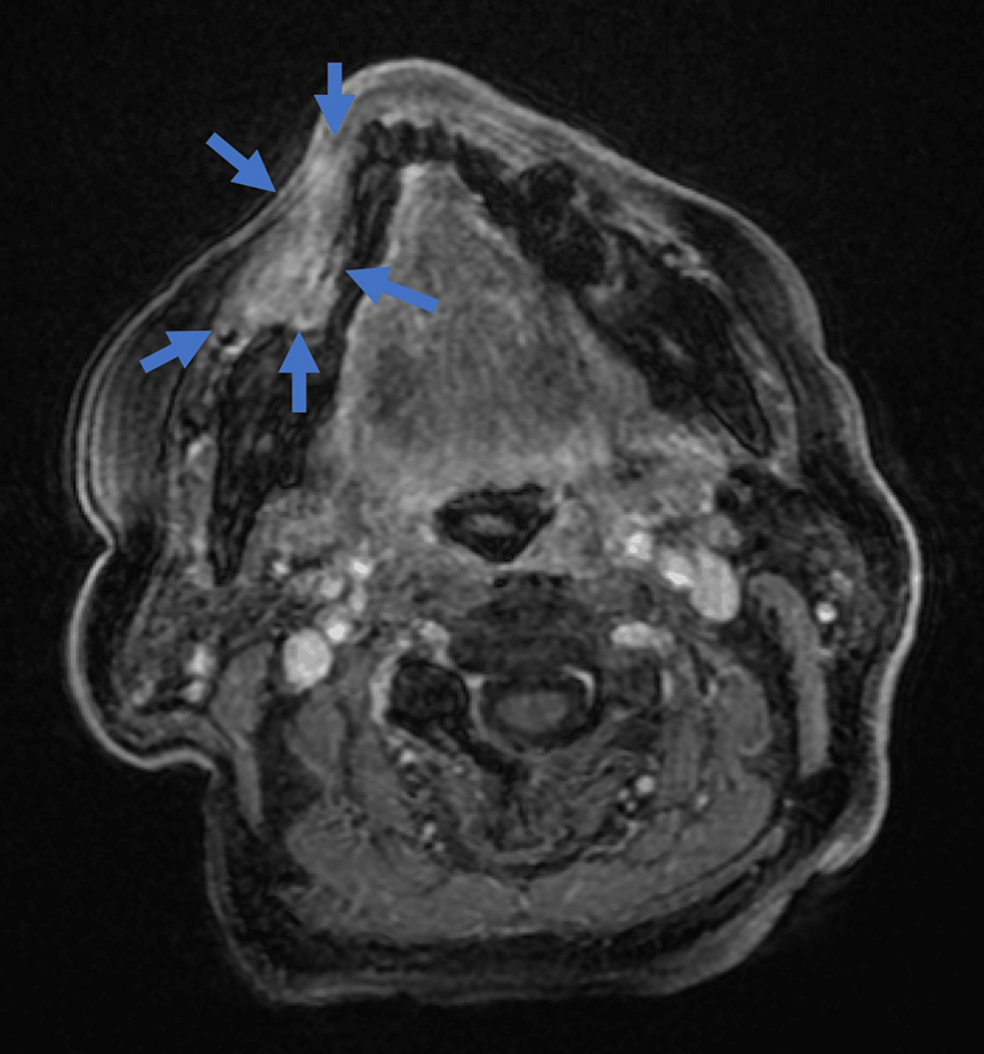
MRI Gadolinium-enhanced fat-suppressed T1-weighted images revealed a mass lesion in the right buccal mucosa with contrast enhancement.

MRI scans using gadolinium-enhanced, fat-suppressed T1-weighted images (T1WI) identified a mass lesion in the right buccal mucosa with notable contrast enhancement (Figure 4).

**Figure 5:**
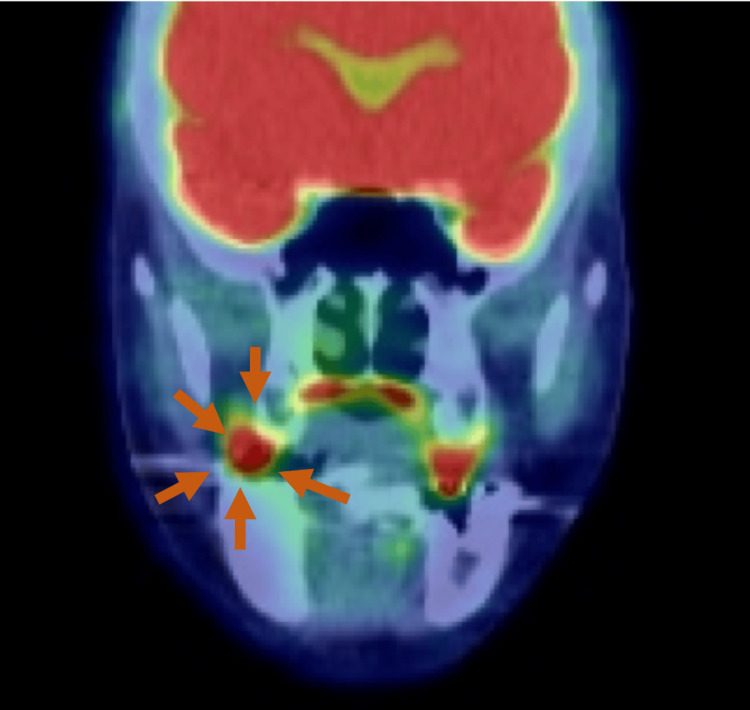
PET-CT Significant uptake noted in the primary tumor, with no suspicious accumulations in cervical lymph nodes or elsewhere in the body.

PET-CT scans demonstrated significant uptake in the primary tumor, without suspicious accumulations in cervical lymph nodes or distal sites (Figure 5).

Neck ultrasonography revealed no indications of metastasis to cervical lymph nodes, supporting an initial diagnosis of right buccal mucosal cancer.

**Figure 6:**
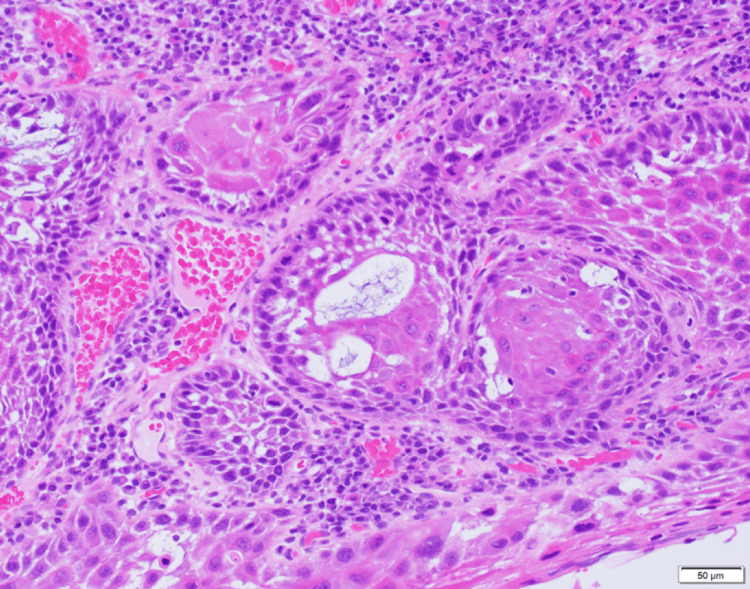
Histopathological Findings at the Time of Initial Surgery (Hematoxylin and Eosin Staining) Polygonal squamous cell carcinoma cells were identified. The tumor exhibited superficial invasion into the interstitium, with no infiltration into the deeper tissues. Neither lymphatic nor vascular invasion was observed.

The treatment commenced with a biopsy, confirming the presence of squamous cell carcinoma in the right buccal mucosa. In July 2020, the patient underwent a partial resection of the buccal mucosa, including the mandibular gingival periosteum, along with the resection of the buccinator muscle and gingival mucosa extending to the periosteum. The postoperative histopathological examination indicated squamous cell carcinoma with clear margins, absent of any spindle cell components (Figure 6). Follow-up care included quarterly CT scans.

**Figure 7:**
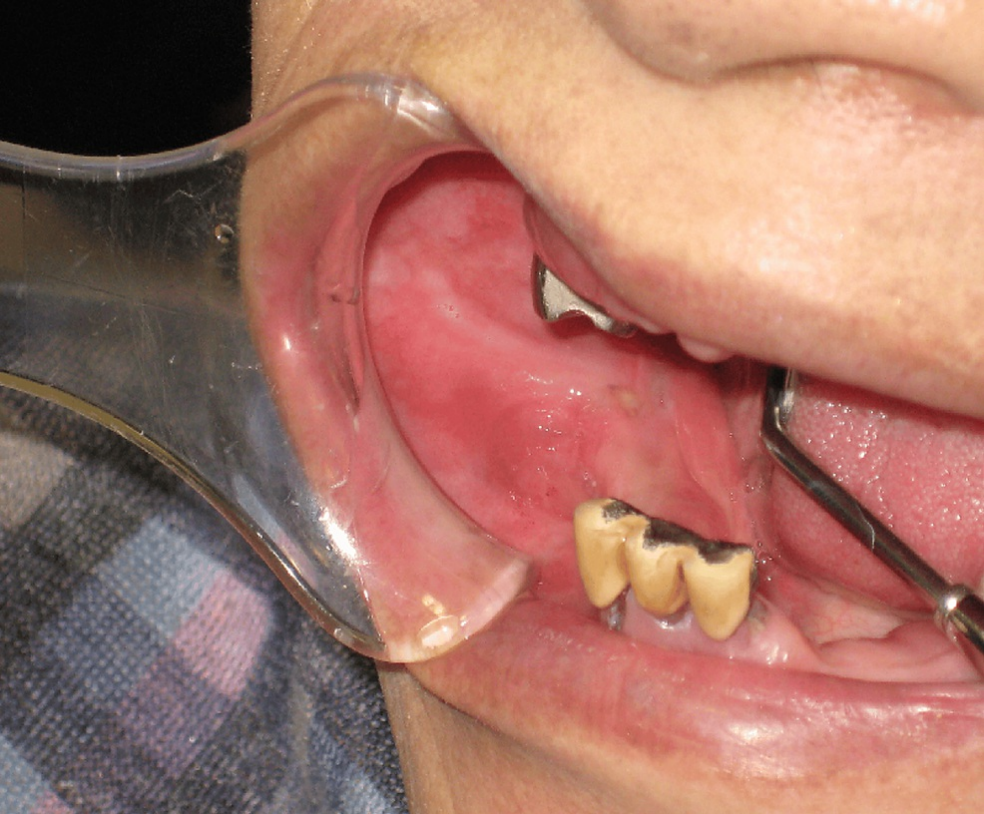
Follow-up Findings No recurrence was observed in the oral cavity.

**Figure 8:**
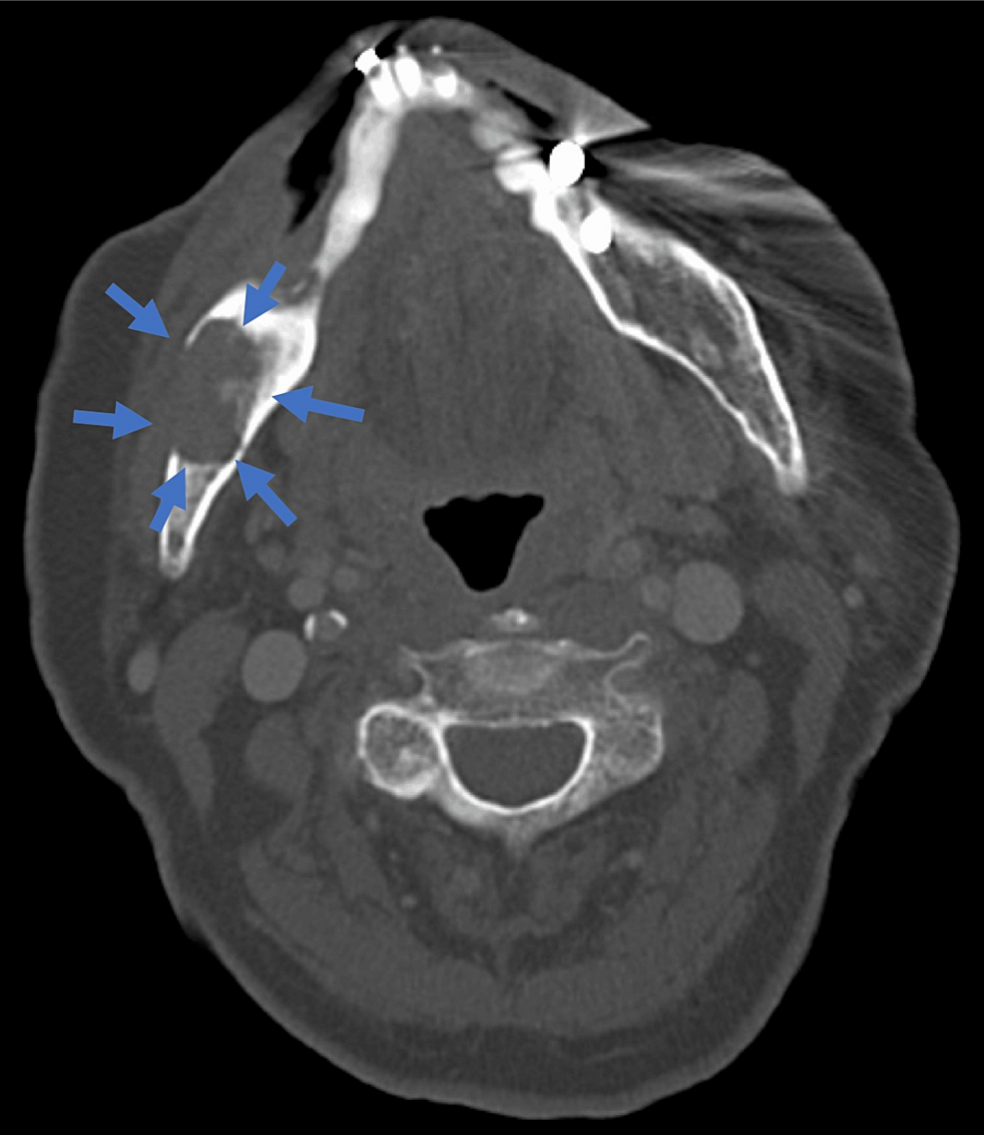
CT scan A large, contrast-enhanced lesion was observed in the right mandible, with cortical bone destruction evident on both the buccal and lingual aspects. No abnormalities were noted in the neck or distant organs.

**Figure 9:**
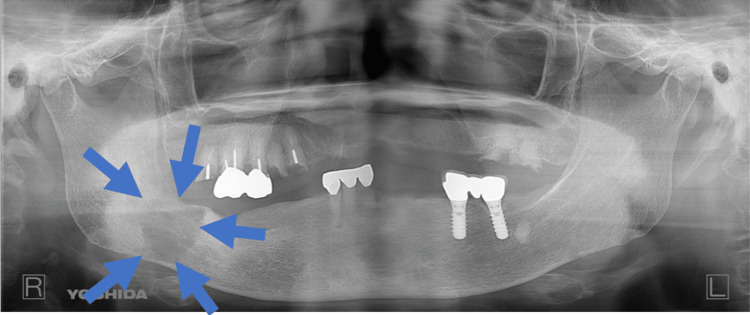
Panoramic X-ray Panoramic X-Ray in the Event of Recurrence or New Lesion A radiolucent lesion, approximately 32x25 mm in size, was observed in the body of the right mandible.

**Figure 10:**
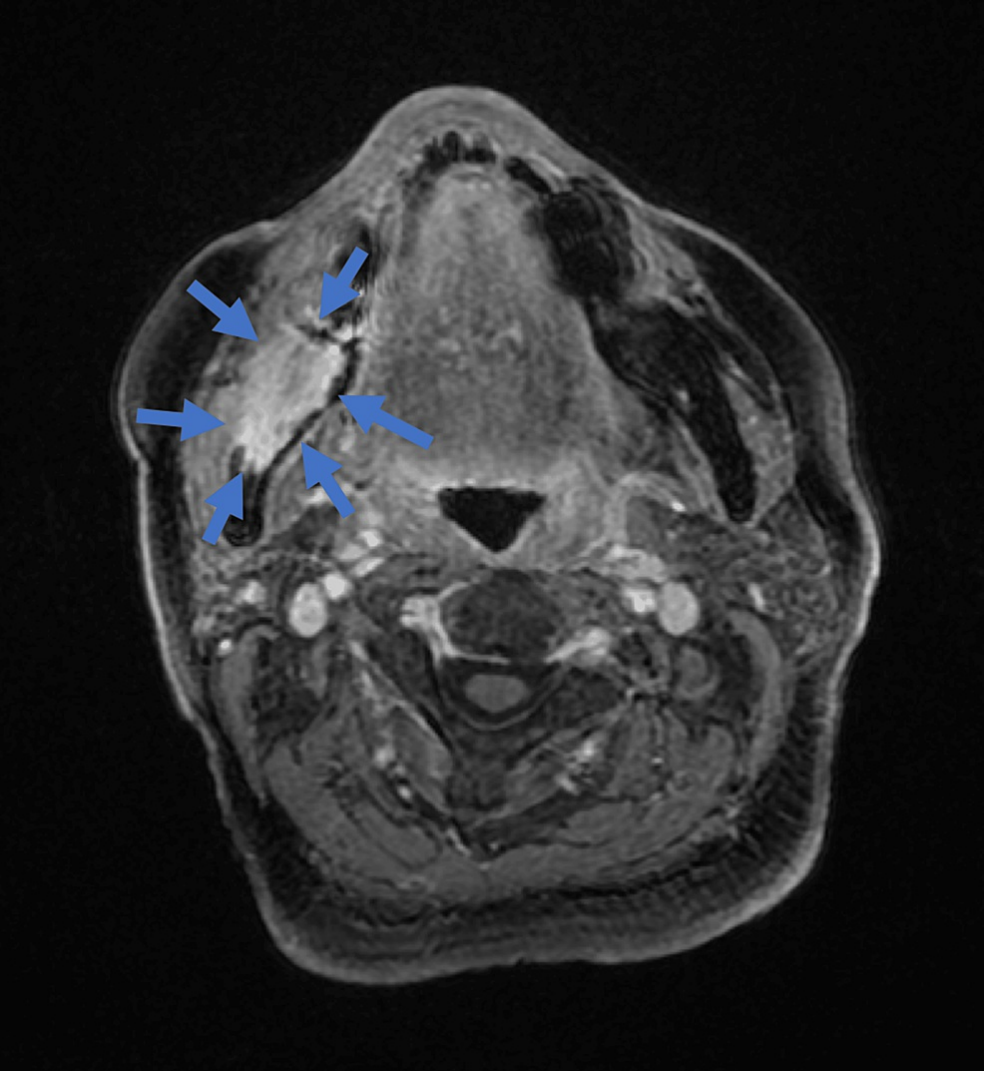
MRI Gadolinium-enhanced fat-suppressed T1-weighted imaging of the right mandible revealed a large lesion with contrast enhancement.

In November 2021, no recurrence was observed in the oral cavity (Figure 7). However, new findings in CT scans indicated bone destruction in the right mandible with enhanced contrast (Figure 8). This was corroborated by panoramic X-rays displaying a distinct lesion in the right mandible and MR images confirming a contrast-enhanced mass (Figure 9, 10). PET-CT scans continued to show significant uptake in the primary tumor but no metastatic evidence. Neck ultrasonography results remained unremarkable.

**Figure 11:**
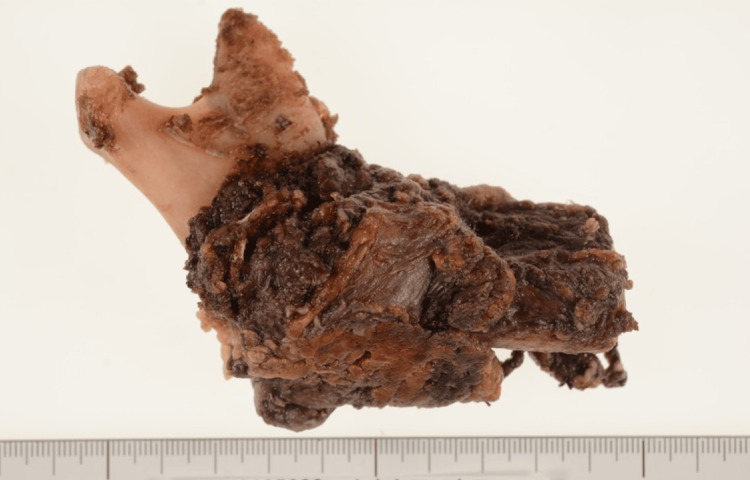
Surgical resection specimen findings in the event of recurrence or new lesion The resection was performed with a margin of at least 10 mm from the tumor, encompassing the submandibular gland, mylohyoid muscle, and sublingual gland on the lingual side, and the masseter and buccinator muscles on the buccal side, to account for potential extra-mandibular invasion.

Owing to severe scar contracture, a re-biopsy under anesthesia was conducted, leading to a revised histopathological diagnosis of spindle cell sarcoma. This necessitated a hemimandibular resection, including the excision of adjacent muscles (Figure 11). The patient exhibited a favorable recovery 12 months post-surgery, with no recurrence or metastatic signs.

**Figure 12:**
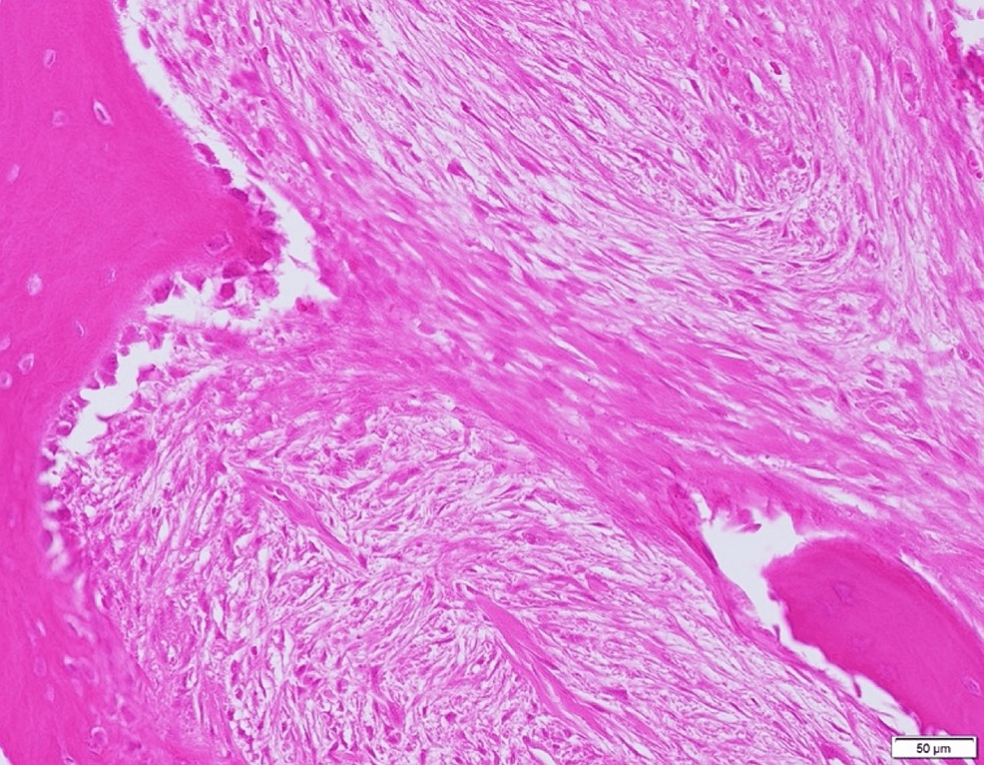
Histopathological findings (Hematoxylin and Eosin staining) Interstitial fibrosis was observed just below the epithelium, with atypical spindle cells present within this region. Furthermore, proliferation of spindle-shaped cells was noted in the mandibular bone, leading to trabecular destruction.

**Figure 13:**
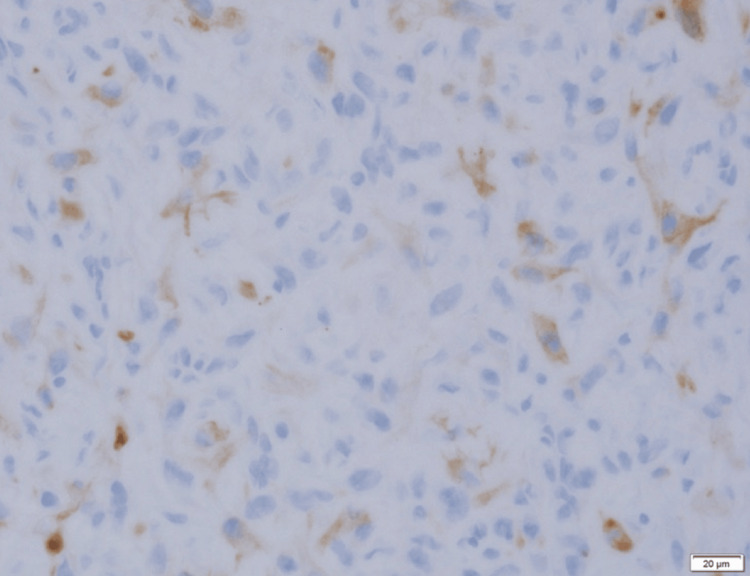
Histopathological findings (Cytokeratin AE1/AE3) Cytokeratin AE1/AE3 exhibited positive staining.

**Figure 14:**
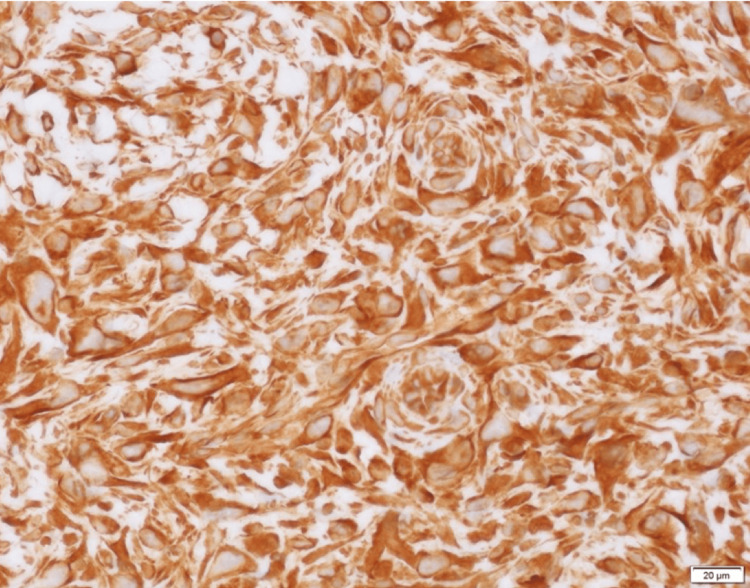
Histopathological findings (Vimentin) Vimentin showed positive staining.

Histopathological analysis revealed interstitial fibrosis beneath the epithelium and atypical spindle cells surrounding it, with these cells proliferating in the mandible and disrupting trabeculae (Figure 12). Immunohistochemical analysis showed partial positivity for cytokeratin (AE1/AE3) and diffuse positivity for vimentin (Figure 13, 14). While tumor cells invaded the buccinator muscle, there was no invasion into the suprahyoid muscle group. There was evidence of invasion into the mandibular canal but without nerve invasion or compression. The surgical margins were clear, confirming a final diagnosis of spindle cell squamous cell carcinoma (SpC-SCC).

## Discussion

Spindle Cell Squamous Cell Carcinoma (SpC-SCC) represents a relatively uncommon neoplasm among oral cancers. Histopathologically, SpC-SCC is characterized by the coexistence of squamous cell carcinoma cells and sarcomatoid spindle cells, as identified through Hematoxylin and Eosin (HE) staining. Immunohistochemical analyses have demonstrated that these spindle cells express markers indicative of both epithelial and mesenchymal lineages [7]. Typically, the squamous epithelial component of this tumor is observed primarily at the base [8], and due to the limited tissue sample size typically obtained from biopsy specimens, it becomes challenging to concurrently capture both squamous cell carcinoma cells and spindle cells, thus complicating definitive diagnosis from such specimens. In the presented case, trismus resulting from postoperative scar contracture impeded the collection of an adequate tissue sample during an outpatient biopsy under local anesthesia.

Consequently, a rapid intraoperative diagnosis under general anesthesia was necessitated. Despite an initial diagnosis of spindle cell sarcoma, further immunohistochemical examination of the resected specimen, which revealed partial positivity for cytokeratin (AE1/AE3) and diffuse positivity for vimentin, led to a conclusive diagnosis of SpC-SCC. These findings underscore the inherent difficulties in diagnosing this condition using biopsy specimens alone.

The etiology of SpC-SCC is multifaceted, ranging from idiopathic origins to secondary transformations post-radiation therapy or chemotherapy [3]. In this case, it is plausible that the tumor originated either idiopathically from the scar tissue at the surgical site or as a secondary transformation of a recurrent tumor at the margin. Considering the lack of evidence of intra-mandibular lesions in imaging studies and a disease-free interval of one-year post-surgery, the presence of SpC-SCC at the time of initial treatment seems improbable. It is more likely that the SpC-SCC emerged either as a secondary recurrence or idiopathically postoperatively.

In instances where the lesion is a secondary recurrence, the histopathological analysis of the specimen from the initial surgery indicated negative margins and no lymphovascular invasion. This suggests that undetected cancer cells could have remained within the tissue, undergoing transformation during proliferation. Although numerous reports exist of SpC-SCC transformations following chemotherapy or radiotherapy [1-5], cases have been diagnosed in surgical specimens post-preoperative chemotherapy [2] and post-chemoradiotherapy or radiation therapy [1-5]. The timing of such transformations varies, with diagnoses occurring immediately post-preoperative chemotherapy or between 6 months to 4 years after initial treatment in recurrent lesions [1-5]. For instance, a patient diagnosed with SpC-SCC four years after a partial glossectomy suggests the transformation of residual tumor cells at the wound margin [6]. However, in our case, the absence of jawbone infiltration during initial treatment and the tumor's location in the scar area imply an idiopathic origin or adjacent organ involvement.

Considering the idiopathic nature of our case, with no continuity with the oral mucosa or metastasis from other organs, the diagnosis aligns with intraosseous carcinoma per the WHO classification [9]. Intraosseous SpC-SCC is exceedingly rare, with only four cases, including our own, reported in Japan [10-12]. The clinical and histopathological findings suggest that this case's etiology could be idiopathic and secondary. Regardless of its origin, this represents an extremely rare occurrence.

Conventionally, radical resection is the preferred treatment for SpC-SCC, similar to standard oral squamous cell carcinoma (SCC)[13]. However, SpC-SCC tends to have a higher propensity for local recurrence and distant metastasis compared to SCC. In unresectable recurrence or distant metastasis cases, radiotherapy and chemotherapy are administered following SCC protocols. Nonetheless, instances of radiation resistance and the absence of established pharmacotherapy have been reported [14,15].

Prognostically, Ellis et al. observed that among 59 head and neck SpC-SCC cases [16], 25 succumbed within two years. The 5-year survival rate for oral SCC stands at 66.5% [17], indicating a notably poorer prognosis for SpC-SCC. For intraosseous SpC-SCC, limited data exists, but Zwetyenga et al. reported a 4-year survival rate of 39.9% [18], suggesting a grim prognosis for intraosseous SpC-SCC.

## Conclusions

This study documents a rare case where buccal mucosal tumor resection, including the mandibular periosteum, was followed by the emergence of SpC-SCC within the mandibular scar 1 year and 4 months post-surgery.
